# Clinical features of ProMisE groups identify different phenotypes of patients with endometrial cancer

**DOI:** 10.1007/s00404-021-06028-4

**Published:** 2021-03-23

**Authors:** Antonio Raffone, Antonio Travaglino, Olimpia Gabrielli, Mariacarolina Micheli, Valeria Zuccalà, Giovanna Bitonti, Caterina Camastra, Valentina Gargiulo, Luigi Insabato, Fulvio Zullo

**Affiliations:** 1 Gynecology and Obstetrics Unit, Department of Neuroscience, Reproductive Sciences and Dentistry, School of Medicine, University of Naples Federico II, Naples, Italy; 2 Anatomic Pathology Unit, Department of Advanced Biomedical Sciences, School of Medicine, University of Naples Federico II, Via Sergio Pansini, 5, 80131 Naples, Italy; 3 Pathology Unit, Monaldi Hospital, Naples, Italy; 4 Pathology Unit, Pugliese-Ciaccio Hospital, Catanzaro, Italy; 5 Department of Obstetrics and Gynecology, Magna Grecia University, Catanzaro, Italy; 6 Department of Health Sciences, University of Catanzaro Magna Græcia, Catanzaro, Italy

**Keywords:** Prognosis, Treatment, Endometrium, Risk assessment, Tumor, Tumour, Carcinoma

## Abstract

**Background:**

The Proactive Molecular Risk Classifier for Endometrial Cancer (ProMisE) groups has identified four molecular prognostic groups of endometrial cancer (EC): POLE-mutated (POLE-mt), mismatch repair-deficient (MMR-d), p53-abnormal (p53-abn), p53-wild-type (p53-wt). These groups might have different pathogenesis and risk factors, and might occur in different phenotypes of patients. However, these data are still lacking.

**Objective:**

To provide a clinical characterization of the ProMisE groups of EC.

**Methods:**

A systematic review and meta-analysis was performed by searching seven electronic databases from their inception to December 2020, for all studies reporting clinical characteristics of EC patients in each ProMisE group. Pooled means of age and BMI and pooled prevalence of FIGO stage I and adjuvant treatment in each ProMisE group were calculated.

**Results:**

Six studies with 1, 879 women were included in the systematic review. Pooled means (with standard error) and prevalence values were:

in the MMR-d group, age = 66.5 ± 0.6; BMI = 30.6 ± 1.2; stage *I* = 72.6%; adjuvant treatment = 47.3%;

in the POLE-mt group, age = 58.6 ± 2.7; BMI = 27.2 ± 0.9; stage *I* = 93.7%; adjuvant treatment = 53.6%;

in the p53-wt group, age = 64.2 ± 1.9; BMI = 32.3 ± 1.4; stage *I* = 80.5%; adjuvant treatment = 45.3%;

in the p53-abn group, age = 71.1 ± 0.5; BMI = 29.1 ± 0.5; stage *I* = 50.8%; adjuvant treatment = 64.4%.

**Conclusion:**

The ProMisE groups identify different phenotypes of patients. The POLE-mt group included the youngest women, with the lower BMI and the highest prevalence of stage I. The p53-wt group included patients with the highest BMI. The p53-abn group included the oldest women, with the highest prevalence of adjuvant treatment and the lowest prevalence of stage I. The MMR-d group showed intermediate values among the ProMisE groups for all clinical features.

**Supplementary Information:**

The online version contains supplementary material available at 10.1007/s00404-021-06028-4.

## Introduction

Endometrial cancer (EC) is the most prevalent gynecologic tumor in the western countries [1]. In the last decades, it also increased in incidence and mortality, due to an inaccurate histopathologic-driven management of patients [1–5]. The current histopathologic risk assessment is indeed poorly reproducible, leading to over- or undertreatment of women, and misinterpretations of findings within clinical trials [5, 6].

In 2013, The Cancer Genome Atlas (TCGA) Research Network has identified four novel prognostic groups of EC based on molecular signatures [7]. Due to technical difficulties and costs of sequencing analysis, a novel molecular classifier, the Proactive Molecular Risk Classifier for Endometrial Cancer (ProMisE), has been developed based on immunohistochemistry as surrogate of sequencing [2, 6, 8, 9]. Immunohistochemistry is indeed more diffuse in the clinical practice because it is inexpensive and fast [10–16]. ProMisE classifies ECs in the following four prognostic groups: POLE-mutated (POLE-mt), mismatch repair-deficient (MMR-d), p53-abnormal (p53-abn), p53-wild-type (p53-wt). POLE-mt group includes ECs with the best prognosis and the highest mutational load; this group is characterized by mutations in the exonuclease domain of Polymerase-ε (POLE) and is the only one that can be identified exclusively by sequencing. MMR-d group has intermediate prognosis, high mutational load and microsatellite instability; this group can be identified by deficient immunohistochemical expression of mismatch repair protein (MMR). P53-abn group has the worst prognosis, low mutational load, high somatic copy number alteration rate and TP53 mutation; this group can be identified by abnormal immunohistochemical expression of p53. P53-wt group has good-to-intermediate prognosis, low mutational load, low somatic copy number alteration rate, and absence of a molecular signature; this group is identified by excluding molecular signatures of the other groups [2, 3, 6, 8, 9].

Given the differences in terms of molecular background, histologic characteristics and prognosis [17–22], these groups may be considered as different diseases within endometrial cancer landscape. These different entities might also have different pathogenesis and risk factors, and might occur in different phenotypes of patients. Specific clinical features in each ProMisE group may allow hypothesizing tailored prevention strategies and additional treatments (e.g. bariatric surgery and/or diet in the groups associated with obesity) for the single patient in the era of precision medicine [23]. Moreover, specific clinical characteristics may contribute to the prognosis of the ProMisE groups (e.g. younger age, early FIGO stage and/or more common adjuvant treatment may be associated with better prognosis). Therefore, while prognostic and histopathological features of the ProMisE groups were previously summarized [3, 17], this study aimed to provide a clinical characterization of the ProMisE groups of EC, with regards to age, body mass index (BMI), FIGO stage, and adjuvant treatment.

## Materials and methods

### Study protocol

Methods for each study step (i.e. search strategy, study selection, assessment of risk of bias within studies, data extraction and analysis) were a priori within the study protocol. Each study step was independently completed by two authors (AR, AT). All authors were asked for solution of disagreements. The study was reported according to the Preferred Reporting Item for Systematic Reviews and Meta-analyses (PRISMA) statement [24].

### Search strategy

Search strategy was planned using seven electronic databases (i.e. Web of Sciences, Google Scholar, Scopus, ClinicalTrial.gov, MEDLINE, Cochrane Library and EMBASE) from their inception to December 2020. The following text words were alternatively combined: “ProMisE”; “Proactive Molecular Risk Classifier”; PORTEC”; “TransPORTEC”; “TCGA”; “Atlas”; “genome”; “survival”; “prognosis”; “endometr*”; “tumor”; “tumour”; “neoplas*”; “cancer”; “carcinoma”; “endometrioid”; “adenocarcinoma”; “serous”; “clear cell”; “undifferentiated”; “ultramutated”; “hypermutated”; “ copy number”; “POLE”; “mismatch repair”; “MMR”; “MMR-d”; “MSI”; “microsatellite instability”; “MLH1”; “MSH2”; “MSH6”; “PMS2”; “EPCAM”; “TP53”; “p53”; “tumor protein 53”; “surrogate”; “immunohistochemistry”; “immunohistochemical”; “marker”;”sequencing”. We considered also all references from each full-text screened study.

### Study selection

All peer-reviewed studies reporting clinical characteristics (age, BMI, FIGO stage, adjuvant treatment) of EC patients by each ProMisE group were included in our review. A priori defined exclusion criteria were: case reports, reviews, data not extractable, and studies with patients’ selection based on pathological characteristics of ECs (they were not representative of a real EC population). For studies with overlapping data (i.e. same period of enrollment, study population, institution, and/or findings), if the original data in each study could not be extracted separately, the study with smaller sample size was excluded from the quantitative analysis.

### Data extraction

Data were extracted from the included studies without modification and according to the PICO (Population, Intervention, Comparator, Outcomes) items [24].

“Population” of our study was patients with EC.

“Intervention” (or risk factor) was the MMR-d, POLE-mt or p53-abn group of EC according to the ProMisE.

“Comparator” was not considered because it was not applicable (meta-analysis of prevalence).

“Outcomes” were the means ± standard error of age and BMI, and the prevalence of the FIGO stage I and adjuvant treatment in the ProMisE groups of EC.

In the studies with overlapping patient data, duplicate data were excluded and only original data were considered.

### Assessment of risk of bias within studies

The assessment of risk of bias within studies followed the Methodological Index for Non-Randomized Studies (MINORS) statement [25]. The following six domains related to risk of bias were applicable: (1) aim (if the aim was clearly stated); (2) inclusion of consecutive patients (if all eligible patients during the study period were included); (3) prospective collection of data (if an a priori defined protocol was adopted for data collection); (4) endpoints appropriate to the aim (if outcomes were evaluated according to clearly stated criteria); (5) unbiased assessment of the study endpoint (if two or more authors performed a blind evaluation, re-evaluation or evaluation of study endpoints); (6) follow-up period appropriate to the aim (if the follow-up time was more than 2 years, which is the minimal follow-up period for patients with endometrial cancer).

Each domain was judged by authors as “low risk”, “unclear risk”, or “high risk” of bias if data were “reported and adequate”, “not reported”, or “reported but inadequate”, respectively.

### Data synthesis

Means of age and BMI, and prevalence of FIGO stage I and adjuvant treatment in each ProMisE group of EC were calculated for each included study and as pooled estimate. They were graphically reported on forest plots, with 95% confidence interval (CI).

The inconsistency index *I*^2^ was used to assess statistical heterogeneity among included studies, as previously described [26, 27]. Heterogeneity was considered null for *I*^2^ = 0, minimal for *I*^2^ < 0.25, low for *I*^2^ < 0.50, moderate for *I*^2^ < 0.75 and high for *I*^2^ ≥ 0.75.

All analyses were performed by adopting the random effect model of DerSimonian and Laird.

Data analysis was performed by Review Manager 5.3 (Copenhagen: The Nordic Cochrane Centre, Cochrane Collaboration, 2014) and Comprehensive Meta-Analysis (Biostat,14 North Dean Street, Englewood, NJ 07631, USA).

## Results

### Study selection

Electronic searches identified 5,784 studies. 895 studies remained after duplicates removal. 52 studies remained after title screening. 21 studies were evaluated for eligibility after abstracts screening. Lastly, six studies were included in the systematic review and five studies in the meta-analysis [2, 6, 8, 9, 28, 29].

Figure S1 graphically shows the study selection process.

### Study and patients’ characteristics

Our qualitative analysis included a total of 1,879 women with EC from retrospective cohorts. Mean age of patients ranged from 42.9 ± 5.6 to 66.9 ± 0.7, and mean BMI ranged from 29 ± 7.7 to 33 ± 1. Overall, prevalence of Stage I and adjuvant treatment was 75.1% and 48.8%, respectively. Of total EC, 23.9% were classified in MMR-d, 10% in POLE-mt, 51% in p53-wt, and 15% in p53-abn groups.

Characteristics of the included studies and patients are shown Tables S1 and S2, respectively.

### Risk of bias within studies assessment

All included studies were judged at low risk of bias in all domains, except for “Inclusion of consecutive patients” domain. In particular, four studies were judged at “unclear risk” of bias in this domain because they did not report if all eligible patients during the study period were included [2, 6, 9, 28]. Furthermore, Britton et al. performed a patient’s selection based on age, only including women aged 49 or younger [9]. The remaining study was judged at low risk of bias.

Assessment of risk of bias within studies is graphically shown in Figure S2.

### Meta-analysis

All duplicate patient data were excluded from the meta-analysis: the study by Britton et al. was excluded because of an overlap of patients with the other included studies [9]; for the Talhouk 2017 study, only the new patients (“confirmation cohort”) were included in the meta-analysis, while patients overlapping with Talhouk 2015 (“discovery cohort”) were excluded [2, 6]; the study by Kolehmainen et al. was included only in the analysis of FIGO stage, since it reported neither standard errors for age and stage nor data regarding adjuvant treatment [28]. Finally, five studies with 1,622 were included in the meta-analysis of FIGO stage, while four studies with 1,018 women were included in the meta-analyses of age, BMI and adjuvant treatment.

**Fig. 1:**
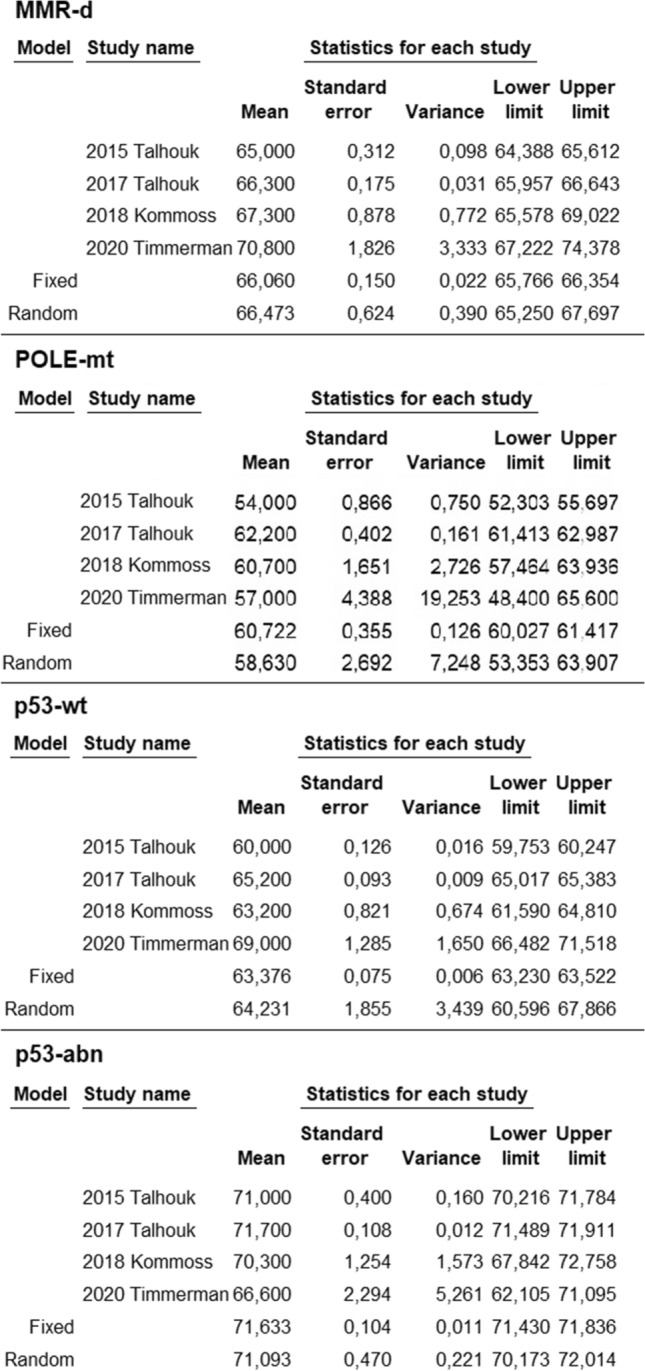
Mean age in ProMisE groups of endometrial cancer for each study and as pooled estimate

Pooled means with standard error of age was 66.5 ± 0.6 (95% CI 65.3–67.7%) in the MMR-d group, 58.6 ± 2.7 (95% CI 53.4–63.9) in the POLE-mt group, 64.2 ± 1.9 (95% CI 60.6–67.9) in the p53-wt group, 71.1 ± 0.5 (95% CI 70.2–72) in the p53-abn group (Fig. 1). Statistical heterogeneity among studies was high for each group, with the exception for the p53-abn group where it was moderate (*I*^2^ = 86.4; *I*^2^ = 96; *I*^2^ = 99.7; *I*^2^ = 66, respectively).

**Fig. 2:**
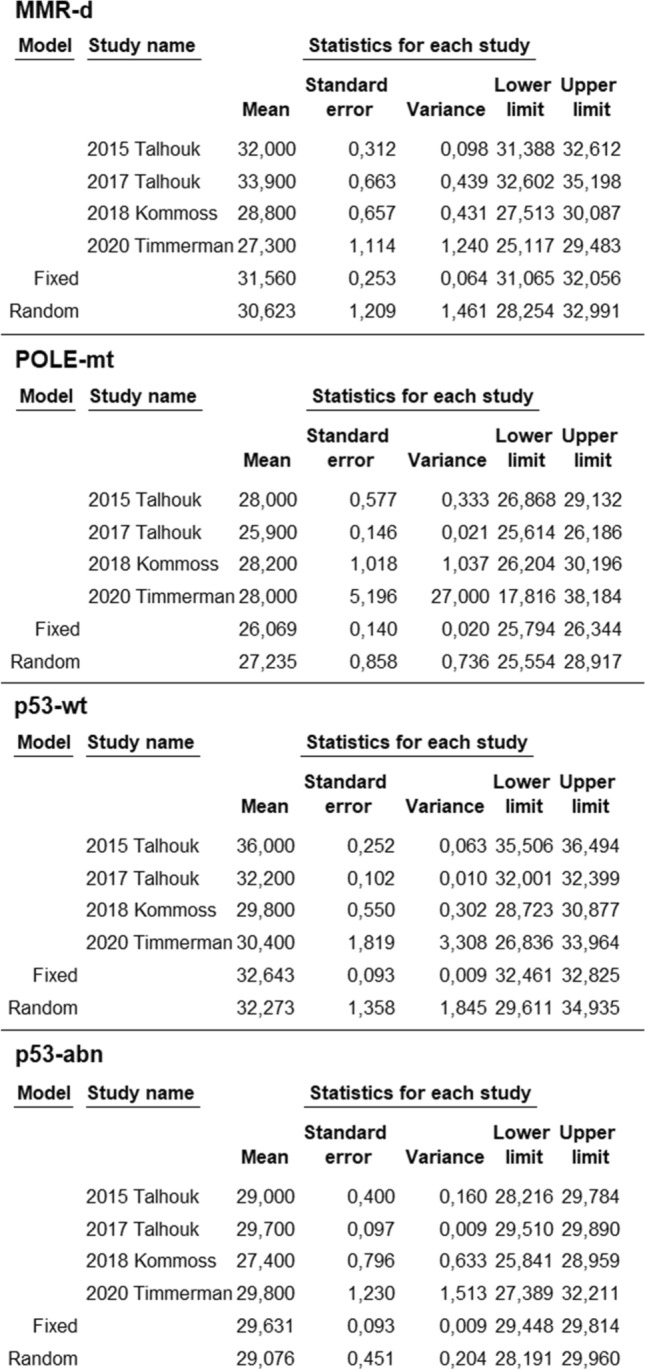
Mean body mass index in ProMisE groups of endometrial cancer for each study and as pooled estimate

Pooled means with standard error of BMI was 30.6 ± 1.2 (95% CI 28.3–33) in the MMR-d group, 27.2 ± 0.9 (95% CI 25.6–29) in the POLE-mt group, 32.3 ± 1.4 (95% CI 29.6–34.9) in the p53-wt group, 29.1 ± 0.5 (95% CI 28.2–30) in the p53-abn group (Fig. 2). Statistical heterogeneity among studies was high for each group (*I*^2^ = 93.6; *I*^2^ = 82.4; *I*^2^ = 98.7; *I*^2^ = 72.4, respectively).

**Fig. 3:**
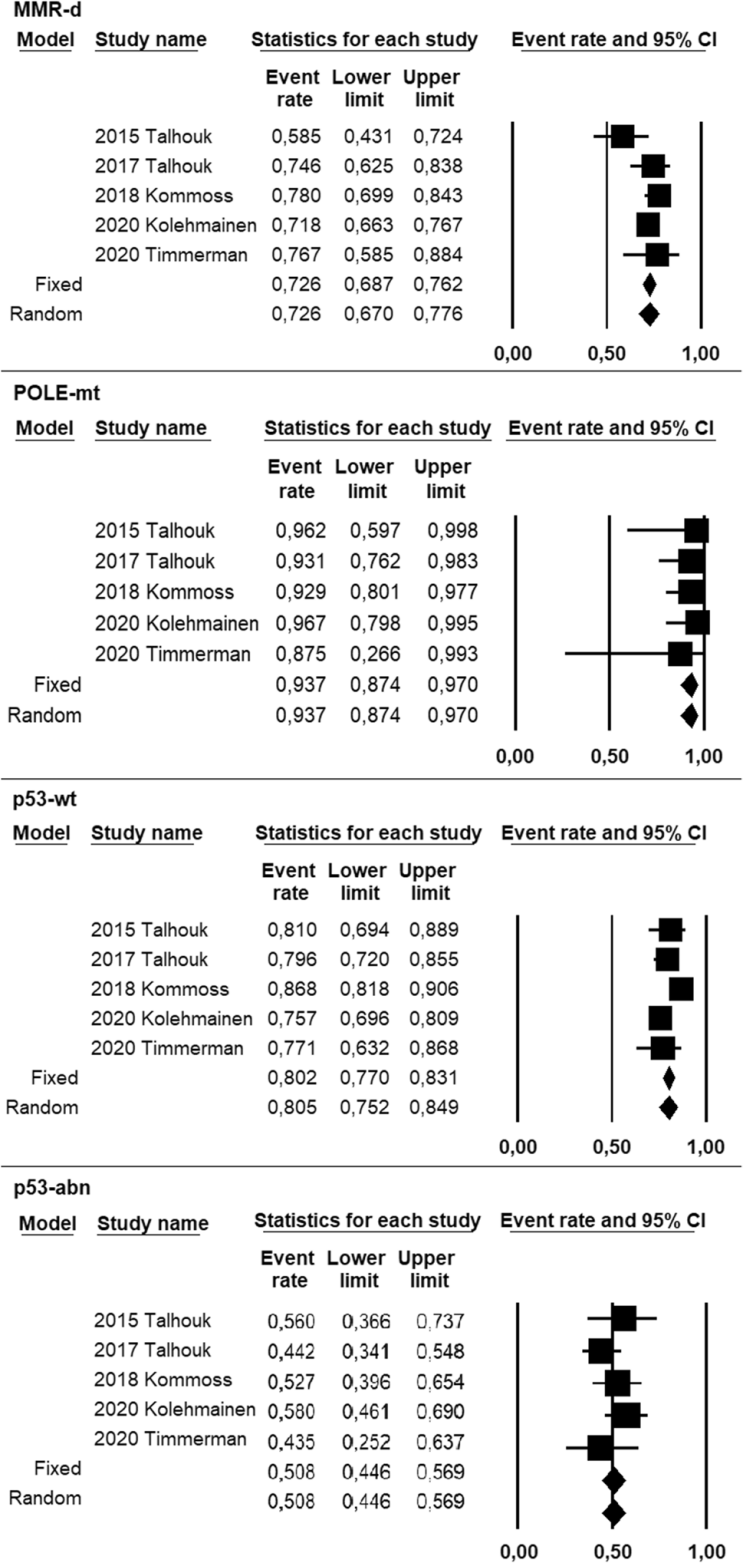
Forest plot of prevalence of FIGO stage I in ProMisE groups of endometrial cancer, including individual study and pooled data

Pooled prevalence of FIGO stage I was 72.6% (95% CI 67–77.6%) in the MMR-d group, 93.7% (95% CI 87.4–97%) in the POLE-mt group, 80.5% (95% CI 75.2–84.9%) in the p53-wt group, 50.8% (95% CI 44.6–56.9%) in the p53-abn group (Fig. 3). Statistical heterogeneity among studies was moderate for the MMR-d group (*I*^2^ = 35.8), null for POLE-mt and p53-abn group (*I*^2^ = 0), and low for the p53-wt group (*I*^2^ = 57.2).

**Fig. 4:**
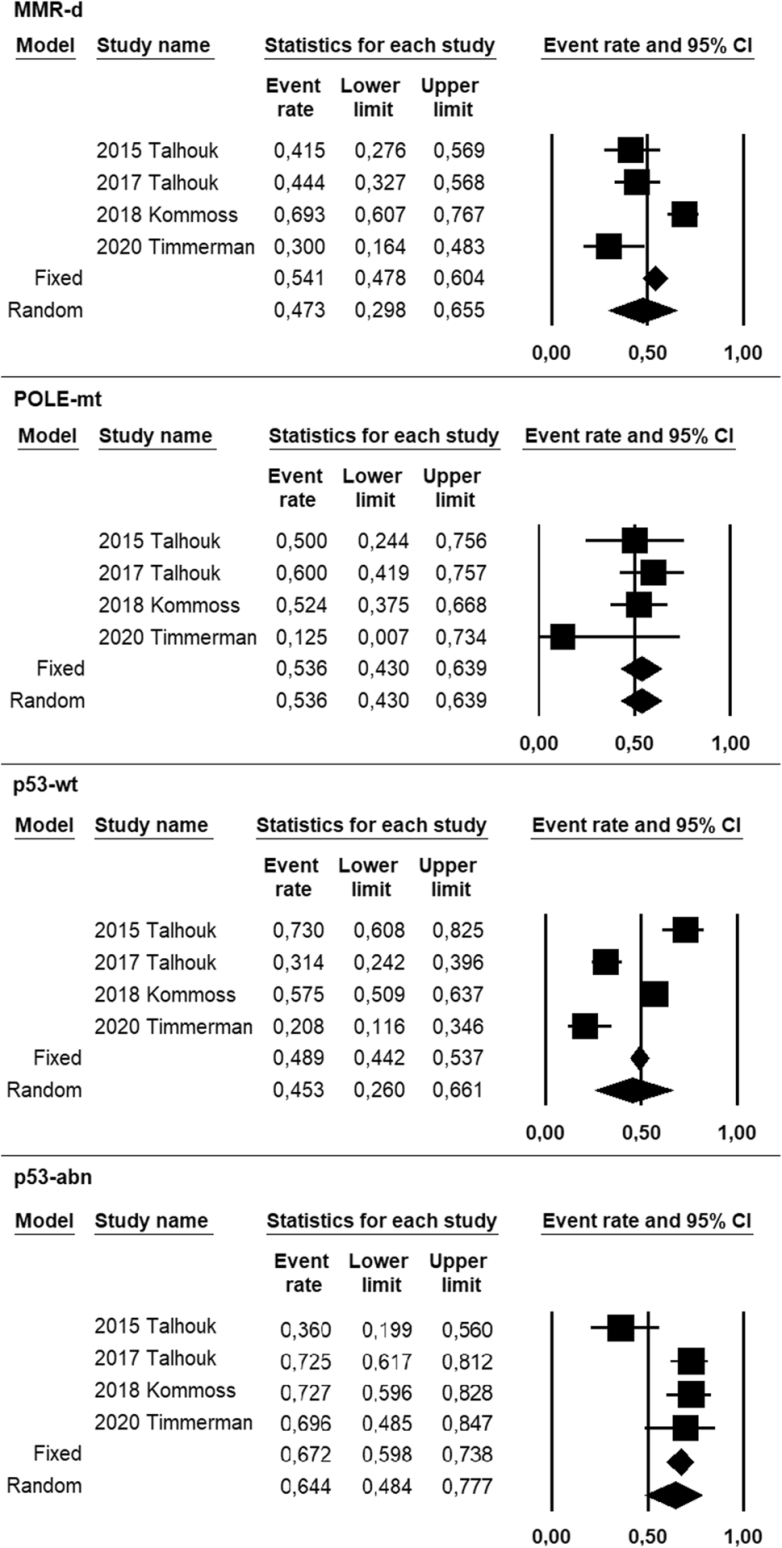
Forest plot of prevalence of adjuvant treatment in ProMisE groups of endometrial cancer, including individual study and pooled data

Pooled prevalence of adjuvant treatment was 47.3% (95% CI 29.8–65.5%) in the MMR-d group, 53.6% (95% CI 43–63.9%) in the POLE-mt group, 45.3% (95% CI 26–66.1%) in the p53-wt group, 64.4% (95% CI 48.4–77.7%) in the p53-abn group (Fig. 4). Statistical heterogeneity among studies was high for each group, with the exception for the POLE-mt group where it was null (*I*^2^ = 86.8; *I*^2^ = 0; *I*^2^ = 93.9; *I*^2^ = 73.8, respectively).

Pooled estimates of means or prevalence of the clinical features in the ProMisE groups are concisely reported in Table S3.

## Discussion

### Main findings and interpretation

This study aimed to provide a clinical characterization of the ProMisE groups of EC to hypothesize possible prevention strategies and additional treatments that may be tailored on the single patient, and to further explain prognosis data across the ProMisE groups. We found that the POLE-mt group included the youngest women, with the lower BMI and the highest prevalence of FIGO stage I. The p53-wt group included patients with the highest BMI and the lowest prevalence of adjuvant treatment. The p53-abn group included the oldest women, with the highest prevalence of adjuvant treatment and the lowest prevalence of FIGO stage I. The MMR-d group showed intermediate values among the ProMisE groups for all clinical features.

### MMR-d group

The intermediate values for all clinical characteristics in this group are in accordance with survival data, which showed an intermediate prognosis [2, 3, 6–8]. In fact, in our previous study, we found that the prognostic value of MMR defect signature was affected by prognostic clinicopathological features. In particular, the difference with the p53-wt group in terms of overall survival, disease-specific survival and progression-free survival became not significant when normalized for clinicopathological factors [3]. Moreover, it has to be shown that the prognostic value of the MMR defect signature may also be affected by the study population. In particular, in early stage endometrioid ECs, it shows an unfavorable prognostic role, while in high-grade ECs it may improve the prognosis [15, 30, 31].

In addition, we found that these patients were obese. Such a finding is in agreement with the hormone-driven pathogenesis of these tumors the first phase of the pathogenesis. In fact, although a MMR defect signature is associated with lower hormone-responsiveness, MMR-d ECs arise from atypical endometrial hyperplasia, which is an hormone-responsive lesion at least in its earliest phases [32, 33]. However, the mean BMI in the MMR-d group was lower compared to p53-wt group; such a difference might be due to the presence of patients with Lynch syndrome, which may develop MMR-d regardless of an initial estrogenic stimulation [32].

### POLE-mt group

The clinical features of the patients in the POLE-mt group seem to outline a specific phenotype: the women in this group were the youngest ones and showed the lowest BMI. These findings might suggest POLE-mt cancers are less “estrogen-related” if compared to the prototypical Bokhman type I EC [7, 34]. In this regard, the high mutational load of POLE-mt ECs might support the onset of estrogen-independent mechanisms [7]. Thus, these carcinomas might also be less responsive to hormonal therapy.

The specific clinical phenotype of the POLE-mt group might also contribute to the favorable prognosis of this group [2, 3, 6–8]. In fact, the younger age of these patients might positively affect overall survival, given the lower likelihood of death, comorbidity, and severe side effect of chemotherapy associated with the young age. The higher prevalence of FIGO stage I (93.3%) may also contribute to the good prognosis. Such prevalence might be due to a low-tumor aggressivity with slow progression, or to more frequent check-ups in younger women with consequently earlier EC diagnosis. The high frequency of adjuvant treatment in POLE-mt EC (53.6%) is likely explained by the high prevalence of high-grade ECs in this group (especially considering that the vast majority is at FIGO stage I), and is a further factor that may improve prognosis [35, 36].

Although age and FIGO stage might have a role in the good prognosis of the POLE-mt group, in our previous study we found that POLE mutation appeared as the molecular signature least affected by other prognostic clinicopathological factors [3]. For this reason, the fact that more than 50% of these women currently undergo adjuvant treatment may constitute an overtreatment.

### p53-wt group

Patients in this group were obese and showed the highest BMI among all groups. This would support the estrogen-driven pathogenesis making inroads to possible prevention strategy based on diet and bariatric surgery for patients with severe obesity. The high BMI, the good-to-intermediate prognosis and the high prevalence of low-grade endometroid carcinomas in this group reflect the prototypical type I EC according to the Bokhman’s classification [34]. Patient age and stage I prevalence were intermediate. These findings may contribute to the good-to-intermediate prognosis in these patients.

### p53-abn group

The p53-abn group appears to embody a specific phenotype of patient, including the oldest women among the ProMisE groups. Such feature is consistent with the high prevalence of serous carcinoma, which arises in a background of atrophic endometrium [7]. Moreover, these patients were non-obese and with the lowest prevalence of FIGO stage I (48.8%). These characteristics, along with the high prevalence of serous histotype, make these cancers assimilable to the type II endometrial cancer of Bokhman’s classification [42]. The highly unfavorable clinical profile of the p53-abn group likely contribute to the poor prognosis of this group. Indeed, in our previous study we found that unfavorable clinicopathological factors sensibly worsened the prognosis of the p53-abn group [3]. However, we also found that the unfavorable prognostic value of the p53-abn signature remained significant even after adjusting for other clinicopathological factors. In this regard, the prognosis remains the worst one although most EC of this group (64.4%) undergo adjuvant treatment.

### Strengths and limitations

To the best of our knowledge, this study may be the first systematic review and meta-analysis to provide a clinical characterization of the ProMisE groups of EC. This study hypothesized that the ProMisE groups might benefit from specific prevention strategies and additional treatments (e.g. diet, bariatric surgery, hormonal therapy) to be tailored on the single patient. Moreover, the provided characterization may be useful to further explain prognosis data across the ProMisE groups. The overall quality of the included studies is very high, given that almost all the domains related to risk of bias were judged at low risk of bias and no one was considered at high risk.

The low number of included studies might be a limitation of our meta-analysis. Nonetheless, the presented data were devoid of patient overlap due to the exclusion of all duplicate data.

## Conclusion

The clinical characterization of ProMisE groups of EC shows that molecular signatures were associated with different phenotypes of patients. The POLE-mt group includes the youngest women, with the lowest BMI and the highest prevalence of FIGO stage I. The p53-wt group includes patients with the highest BMI. The p53-abn group includes the oldest women, with the highest prevalence of adjuvant treatment and the lowest prevalence of FIGO stage I. The MMR-d group showed intermediate values among the ProMisE groups for all clinical features. The clinical characterization of these groups may suggest different pathogenetic mechanisms and may also contribute to explain the prognostic data of the ProMisE groups, with potential impact on the patient management.

## Supplementary Information

Below is the link to the electronic supplementary material.

Supplementary file1 (PNG 11 KB)

Supplementary file2 (PNG 5 KB)

Supplementary file3 (PNG 6 KB)

Supplementary file4 (DOCX 14 KB)

Supplementary file5 (DOCX 17 KB)

Supplementary file6 (DOCX 14 KB)
