# Supplementary figures and images for: Clinical features of ProMisE groups identify different phenotypes of patients with endometrial cancer
Source: Arch Gynecol Obstet. 2021 Mar 23;303(6):1393–400. doi: 10.1007/s00404-021-06028-4 (PMC8087601; doi:10.1007/s00404-021-06028-4)

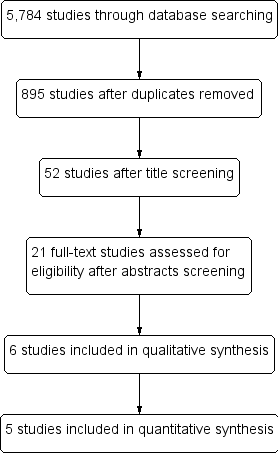

Supplement: Supplementary file 1 — Supplementary file1 (PNG 11 KB) [file 404_2021_6028_MOESM1_ESM.png]

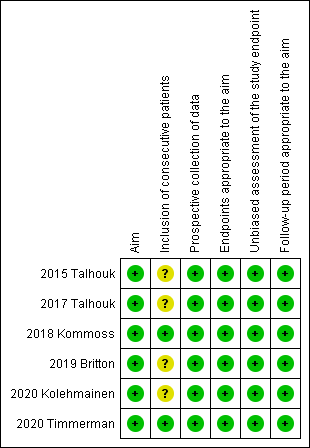

Supplement: Supplementary file 2 — Supplementary file2 (PNG 5 KB) [file 404_2021_6028_MOESM2_ESM.png]

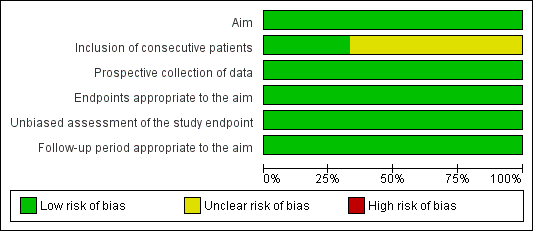

Supplement: Supplementary file 3 — Supplementary file3 (PNG 6 KB) [file 404_2021_6028_MOESM3_ESM.png]
