# Supplementary material for: Clinical features of ProMisE groups identify different phenotypes of patients with endometrial cancer
Source: Arch Gynecol Obstet. 2021 Mar 23;303(6):1393–400. doi: 10.1007/s00404-021-06028-4 (PMC8087601; doi:10.1007/s00404-021-06028-4)
Supplement: Supplementary file 4 — Supplementary file4 (DOCX 14 KB) [file 404_2021_6028_MOESM4_ESM.docx]

**Supplementary Table 1. Characteristics of the included studies**

| **STUDY** | **PERIOD OF INITIAL DIAGNOSIS** | **SETTING** | **COUNTRY** | **TYPE OF COHORT** |
| --- | --- | --- | --- | --- |
| **2015 Talhouk** | 2002 - 2009 | Vancouver General Hospital | Canada | Retrospective cohort |
| **2017 Talhouk** | 1983-2013 | Vancouver General Hospital | Canada | Retrospective cohort |
| **2018 Kommoss** | 2003 - 2013 | Tubingen University Women’s Hospital | Germany | Retrospective cohort |
| **2019 Britton** | 1983-2014 | Vancouver General Hospital  Tubingen University Women’s Hospital | Canada  Germany | Retrospective cohort |
| **2020 Kolehmainen** | 2007- 2012 | Helsinki University Hospital | Finland | Retrospective cohort |
| **2020 Timmerman** | 2017-2019 | University Hospitals Leuven | Belgium | Retrospective cohort |
