# Supplementary material for: Clinical features of ProMisE groups identify different phenotypes of patients with endometrial cancer
Source: Arch Gynecol Obstet. 2021 Mar 23;303(6):1393–400. doi: 10.1007/s00404-021-06028-4 (PMC8087601; doi:10.1007/s00404-021-06028-4)
Supplement: Supplementary file 5 — Supplementary file5 (DOCX 17 KB) [file 404_2021_6028_MOESM5_ESM.docx]

**Supplementary Table 2.** **Clinical features of patients with endometrial cancers by each included study.**

| **STUDY** | **Sample size**  **n (%)** | | | | | **AGE**  **means + SD** | **BMI**  **means + SD** | **STAGE**  **n (%)** | | **Adjuvant treatment**  **n (%)** |
| --- | --- | --- | --- | --- | --- | --- | --- | --- | --- | --- |
|  | **Total** | **MMR-d** | **POLE-mt** | **P53-wt** | **P53-abn** |  |  | **I** | **II-IV** |  |
| **2015 Talhouk** | 143 | 41  (29) | 12  (9) | 63  (45) | 25  (18) | 63 ± 1 | 33 ± 1 | 102  (71) | 41  (29) | 79  (56) |
| **2017 Talhouk** | 319 | 64  (20) | 30  (9) | 139  (44) | 86  (27) | 66.9 ± 0.7 | 31.3 ± 1.2 | 221  (70.2) | 94  (29.8) | 147  (47.4) |
| **2018 Kommoss** | 452 | 127  (28) | 42  (9) | 228  (50) | 55  (12) | 65.0 ± 11.5 | 29 ± 7.7 | 365  (80.8) | 87  (19.2) | 281  (62.2) |
| **2019 Britton** | 257 | 48  (19) | 34  (13) | 164  (64) | 11  (4) | 42.9 ± 5.6 | 31.6 ± 10.2 | 192  (78) | 54  (22) | 65  (43.3) |
| **2020 Kolehmainen** | 604 | 287  (48) | 30  (5) | 218  (36) | 69  (11) | - | - | 440  (73) | 164  (27) | - |
| **2020 Timmerman** | 104 | 30  (28.8) | 3  (2.3) | 48  (46.2) | 23  (22.1) | 68.5 ± 9.8 | 29.2 ± 9.5 | 73  (70.2) | 31  (29.8) | 35  (33.7) |
| **TOTAL** | 1,879 | 597  (31.8) | 151  (8) | 860  (45.8) | 269  (14.3) | - | - | 1,393  (74.1) | 471  (25.1) | 607  (48.8) |
