# Supplementary material for: Clinical features of ProMisE groups identify different phenotypes of patients with endometrial cancer
Source: Arch Gynecol Obstet. 2021 Mar 23;303(6):1393–400. doi: 10.1007/s00404-021-06028-4 (PMC8087601; doi:10.1007/s00404-021-06028-4)
Supplement: Supplementary file 6 — Supplementary file6 (DOCX 14 KB) [file 404_2021_6028_MOESM6_ESM.docx]

**Supplementary Table 3.** Pooled means or prevalence of clinical features in ProMisE groups of endometrial cancer.

| **Clinical characteristic** | **MMR-d group** | **POLE-mt group** | **p53-wt group** | **p53-abn group** |
| --- | --- | --- | --- | --- |
| **Age (mean ± standard error)** | 66.5 ± 0.6 | 58.6 ± 2.7 | 64.2 ± 1.9 | 71.1 ± 0.5 |
| **BMI (mean ± standard error)** | 30.6 ± 1.2 | 27.2± 0.9 | 32.3 ± 1.4 | 29.1 ± 0.5 |
| **Stage I (%)** | 72.6 | 93.7 | 80.5 | 50.8 |
| **Adjuvant treatment (%)** | 47.3 | 53.6 | 45.3 | 64.4 |
